# Cystic intestinal duplication-induced secondary intussusception with associated incidental Meckel’s diverticulum in an infant: a case report with literature review

**DOI:** 10.3389/fsurg.2025.1629836

**Published:** 2025-07-24

**Authors:** Chuanyang Liu, Meng Shi, Jinhua Jia, Yuexia Bai, Shengwei Luan, Hongzhen Liu, Meng Kong

**Affiliations:** ^1^ Department of Pediatric Surgery, Children’s Hospital Affiliated to Shandong University, Jinan, China; ^2^ Department of Pediatric Surgery, Jinan Children’s Hospital, Jinan, China; ^3^ Department of Gastroenterology, Children’s Hospital Affiliated to Shandong University, Jinan, China; ^4^ Department of Pathology, Children’s Hospital Affiliated to Shandong University, Jinan, China; ^5^ Department of Ultrasound, Children’s Hospital Affiliated to Shandong University, Jinan, China

**Keywords:** intestinal duplication cyst, Meckel's diverticulum, secondary intussusception, pediatric, gastrointestinal malformation

## Abstract

**Background:**

Secondary intussusception in children is associated primarily with organic intestinal pathologies. Intestinal duplication constitutes an uncommon lead point for such cases, while its co-occurrence with an incidentally discovered Meckel's diverticulum represents an exceptionally rare clinical scenario. This report describes an 8-month-old female infant who presented with secondary intussusception initially attributed to a cystic intestinal duplication, with Meckel's diverticulum discovered incidentally during surgical exploration.

**Case presentation:**

An 8-month-old female infant was admitted with recurrent vomiting, intermittent fever, and episodes of intense abdominal pain. Abdominal ultrasound revealed ileocolic intussusception and a cystic mass (3.5 cm × 3.0 cm × 3.0 cm) near the ileocecal junction. After unsuccessful air enema reduction, emergency surgery was performed. During the operation, intussusception was found to be caused by cystic intestinal duplication, which acted as the primary lead point. Notably, a separate Meckel's diverticulum (measuring 2.5 cm × 2.0 cm × 1.5 cm in diameter) was discovered incidentally 30 cm proximal to the ileocecal valve on the antimesenteric border of the ileum, demonstrating no pathological connection to the intussusception. Both lesions were surgically removed. Pathological examination confirmed a cystic intestinal duplication and a Meckel's diverticulum containing ectopic gastric tissue. The patient recovered well postoperatively and showed no recurrence of symptoms over a 30-month follow-up period.

**Conclusions:**

In pediatric patients with secondary intussusception caused by enteric duplication, meticulous intraoperative evaluation following successful reduction is critical to identify concurrent intestinal anomalies, including inverted Meckel's diverticulum—a potential lead point for secondary intussusception. This case highlights the incidental discovery of a coexisting Meckel's diverticulum, which was prophylactically excised despite lacking immediate pathological relevance. Systematic exploration combined with tailored resection strategies ensures definitive resolution of intussusception and long-term complication prevention in such rare dual-pathology presentations.

## 1 Introduction

The telescoping of a bowel segment into an adjacent lumen is the most common cause of intestinal obstruction in infants (1). Although 90% of intussusception cases in children are idiopathic, secondary forms are usually triggered by pathological inducing points, the most common of which is Meckel's diverticulum, followed by intestinal polyps and malignant lymphoma. It is less common in intestinal repetitive cysts, allergic purpura or postoperative factors. Unlike idiopathic cases, secondary intussusception frequently resists nonoperative reduction and mandates surgical intervention to address the underlying pathology. The coexistence of multiple lead points, such as Meckel's diverticulum and intestinal duplication, is exceedingly rare and poses significant diagnostic challenges (2). We herein describe a pediatric surgical case in which intestinal duplication precipitated acute intussusception, with intraoperative identification of a concomitant Meckel's diverticulum through standardized laparotomy protocols. This case bridges a critical knowledge gap by illustrating cystic intestinal duplication as a sole lead point for ileocolic intussusception, demonstrating the essential role of systematic intraoperative exploration in detecting synchronous Meckel's diverticulum, and validating the feasibility of single-stage resection for dual pathologies—underscoring how such rare associations necessitate heightened diagnostic vigilance to prevent missed lesions.

## 2 Case report

### 2.1 General information

An 8-month-old female infant presented to our hospital with complaints of “recurrent vomiting with fever for 2 days and paroxysmal crying for 12 h.” Abdominal ultrasound at the local hospital revealed intussusception, and air enema reduction failed, prompting transfer to our institution. A repeat ultrasound confirmed findings consistent with intestinal duplication cysts combined with intussusception. Past medical history, personal history, and family history were unremarkable.

### 2.2 Physical examination and auxiliary examination

**Figure 1:**
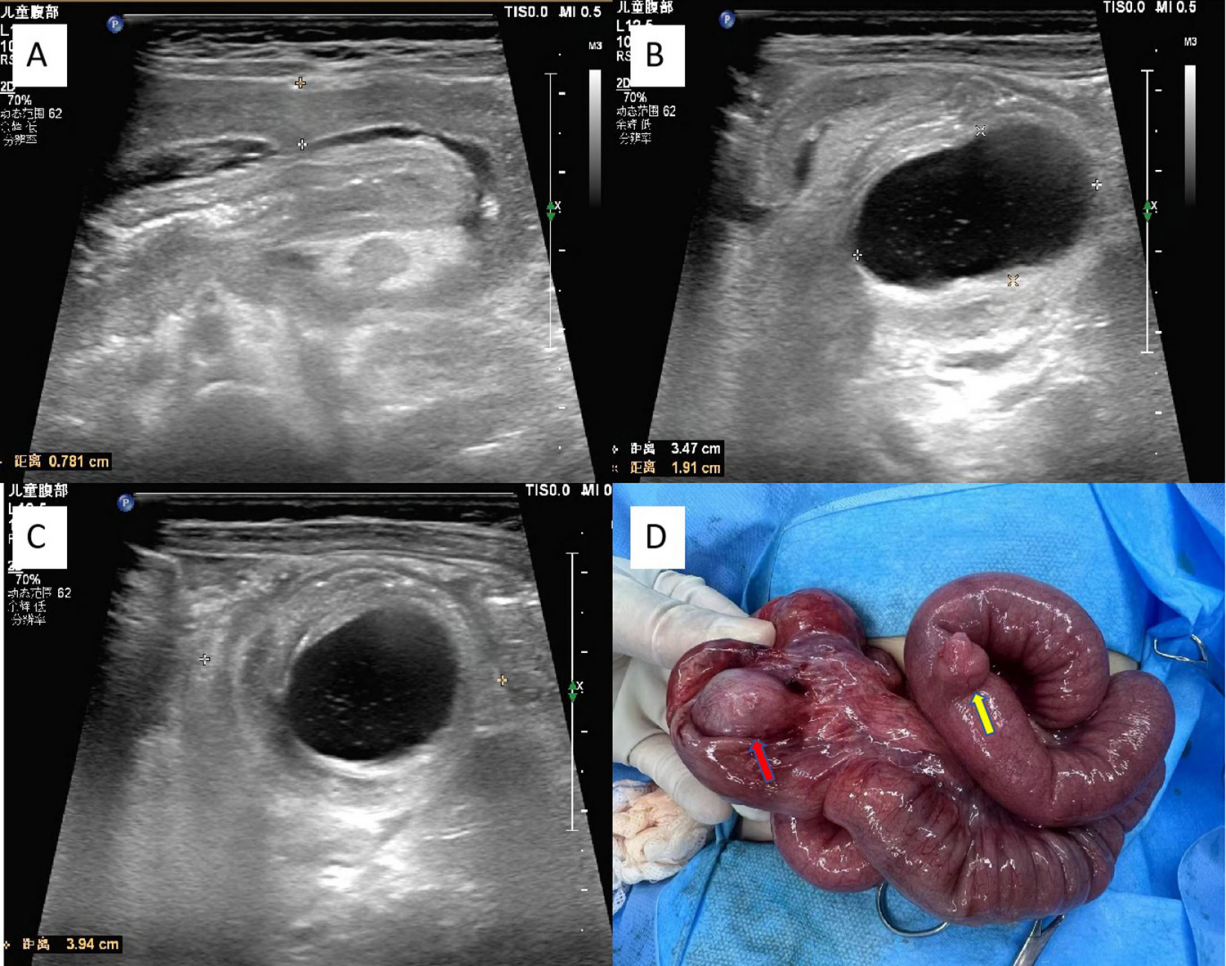
Preoperative color ultrasound and intraoperative images. **(A)** Abdominal ultrasound revealed intussusception, presenting a “sleeve sign” in the longitudinal section. **(B)** Abdominal ultrasound displays image of the cystic long axis of the intussusception head. **(C)** Abdominal ultrasound revealed the cystic long axis of the intussusception head. **(D)** Ileocecal intestinal duplication (Red arrow) and Meckel's diverticulum (Yellow arrow).

Physical examination revealed that the abdomen was slightly distended, with no visible varicose veins in the abdominal wall. The abdominal muscles were slightly tense, with tenderness in the right upper quadrant (+), but there was no rebound tenderness. A sausage-shaped mass, approximately 5 cm × 3 cm in size, firm in texture and mobile, was palpated in the right upper abdomen. The liver and spleen are not palpable below the costal margin. Murphy's sign is (-), and shifting dullness is (-). Bowel sounds occur 6 times per minute. Imaging findings: Ultrasound revealed a target mass (14 cm × 4.2 cm) in the ileocolic region, which was consistent with intussusception. A 3.9 cm × 2.3 cm cystic lesion was noted at the intussusception apex, suggesting an intestinal duplication cyst. Color Doppler revealed diminished blood flow in the affected bowel (Figures 1A–C). Laboratory tests: Blood cell analysis revealed the following results: white blood cell count, 12.03 × 10^9 ^/L; red blood cell count, 3.99 × 10^12 ^/L; and platelet count, 432 × 10^9 ^/L. Blood biochemistry revealed the following: total protein, 59 g/L; globulin, 17 g/L; the ratio of albumin to globulin, 2.5; total bilirubin, 13.4 μmol/L; and direct bilirubin, 5.6 μmol/L.

### 2.3 Surgical procedure

On the basis of the patient's clinical history, physical examination, and imaging features, a preliminary diagnosis of intussusception secondary to an intestinal duplication cyst was established. Given the patient's young age, limited abdominal cavity space, failed air enema reduction, compromised intestinal blood flow, and prolonged clinical course (>48 h), the decision was made to proceed with emergency exploratory laparotomy. The specific operation steps were as follows: After successful anesthesia induction, the patient was placed in the supine position with an F6 urinary catheter placed. The surgical field was prepared via standard antiseptic draping. A right upper transverse abdominal incision (approximately 6 cm in length) was made. The layers-skin, subcutaneous fat, external oblique aponeurosis, internal oblique muscle, transverse abdominis muscle, and peritoneum—were incised sequentially. The intussusception mass (8 cm × 3.5 cm × 3.5 cm) was delivered, confirming ileocolic intussusception. Gentle manual retrograde reduction was performed, revealing preserved bowel viability (localized dark-purple areas regained a normal color after warm saline-soaked gauze application).At the mesenteric border of the terminal ileum, a cystic mass (3.5 cm × 3.0 cm × 3.0 cm) was identified (Figure 1D). The lesion was diagnosed as intestinal duplication on the basis of characteristic intraoperative findings (smooth-walled cyst with a shared muscular layer), and radical resection of the duplication was subsequently performed to ensure complete excision of the pathological segment. Further exploration revealed a diverticulum (2.5 cm × 2.0 cm × 1.5 cm) located 30 cm proximal to the ileocecal valve on the antimesenteric border of the ileum, consistent with Meckel's diverticulum. Radical resection of the diverticulum was subsequently performed, including wedge excision of the adjacent ileal wall with primary anastomosis via interrupted 5-0 absorbable sutures to ensure anatomical integrity. Patency of the anastomosis was confirmed via a water injection test (no leakage was observed). Hemostasis was ensured, and the abdominal wall was closed in layers after verifying instrument counts.

### 2.4 Patient postoperative conditions and pathological findings

**Figure 2:**
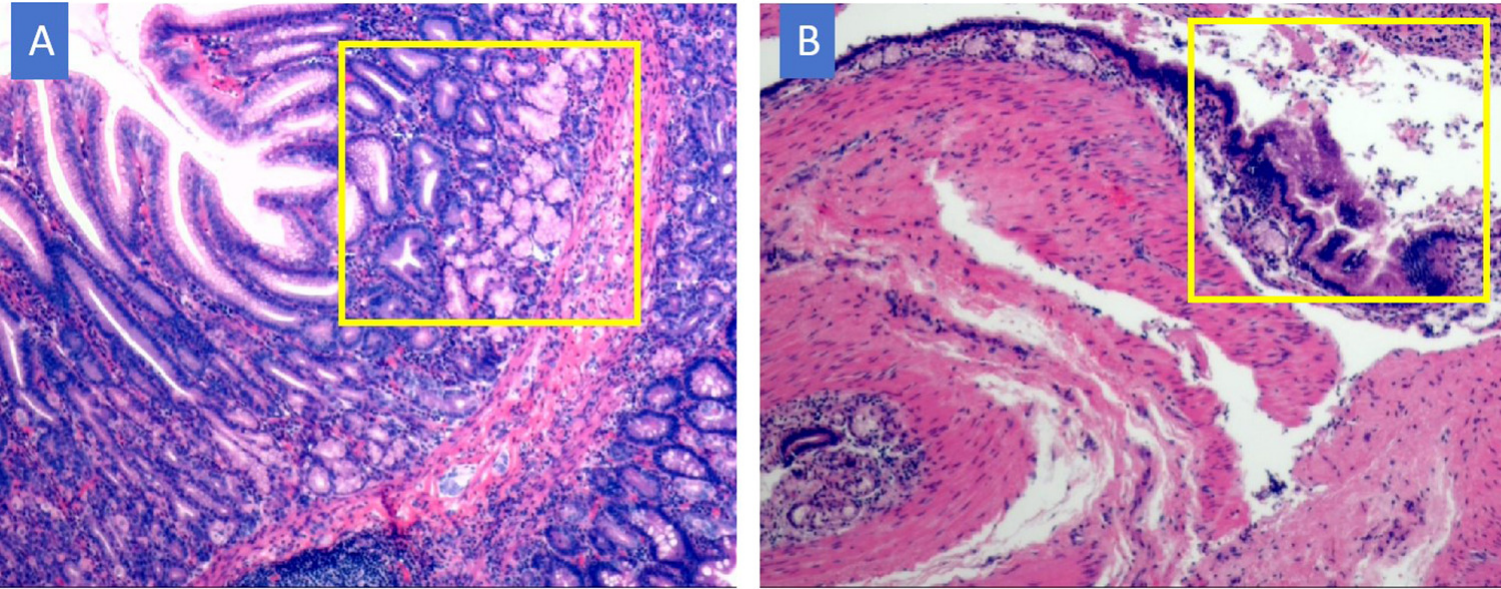
Pathological HE staining results. **(A)** Meckel's diverticulum, The apex of Meckel's diverticulum is lined by ectopic gastric mucosal epithelium; ×40. **(B)** Intestinal duplication. The wall of the duplication cyst has mucosal and muscular layers, with the gastric mucosal epithelium lining; ×40.The yellow-marked area represents ectopic gastric mucosal tissue.

**Figure 3:**
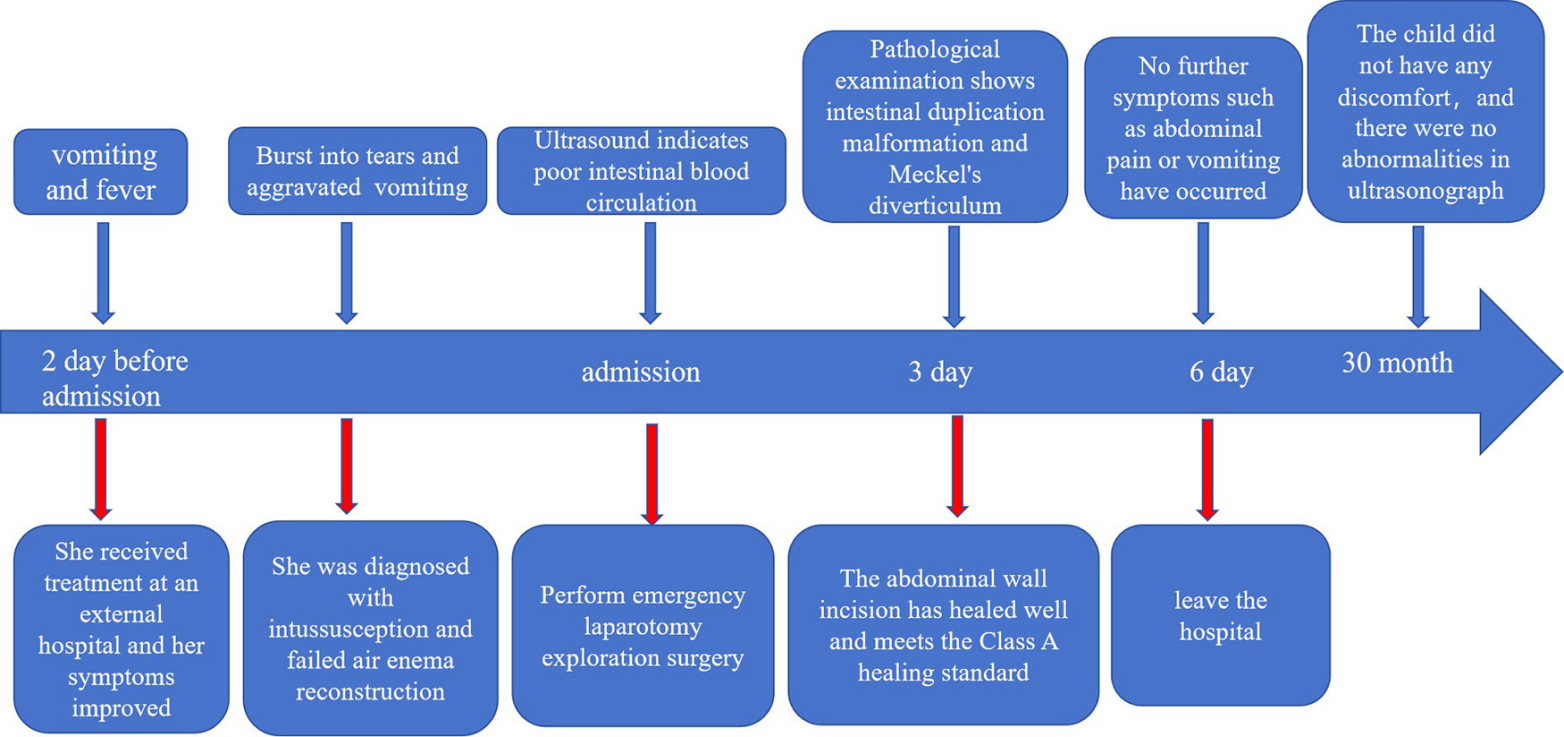
Complete timeline, including diagnosis, surgery, postoperative recovery, and follow-up.

The patient began oral water intake on the first postoperative day, followed by gradual advancement to a regular diet. He was discharged from the hospital on the seventh day following treatment and achieved a full recovery. No complications, such as anastomotic leakage or surgical site infection, were observed during the hospital stay. The resected ileocecal cyst was pathologically confirmed as a disrupted disruption of the intestinal duplication cyst, with its wall partially lined by the gastric mucosal epithelium (Figure 2A). The excised mass located on the antimesenteric border of the ileum, 30 cm proximal to the ileocecal valve, was identified as a Meckel's diverticulum. Histopathological analysis revealed ectopic gastric mucosa and ganglionic cell hyperplasia within the diverticulum, with heterotopic gastric epithelium observed at the apex of the diverticulum (Figure 2B). During the 30-month postoperative follow-up, abdominal ultrasound examinations consistently demonstrated no structural abnormalities in the intestinal tract (Figure 3).

## 3 Discussion

Intestinal duplication malformations and Meckel's diverticulum are both types of embryonic gastrointestinal malformations. Although there are differences in their pathological mechanisms and embryonic origins (the former is caused by a failure in the cavitation of the intestinal canal, whereas the latter is due to the persistence of the yolk sac duct), there is significant overlap in clinical manifestations: painless hematochezia, intestinal obstruction, secondary intussusception, intestinal perforation, and abdominal cavity infections are common clinical presentations. In clinical practice, these two malformations are usually seen as single cases, and there are only a few reports of them occurring together (3, 4). Therefore, while differentiating between these two malformations, one should be alert to the possibility of concurrent occurrence to avoid missed diagnoses.

Intestinal duplication malformations are rare congenital gastrointestinal developmental anomalies, and their anatomical features and key points for differential diagnosis deserve attention. It can occur in any part of the gastrointestinal tract, with the most common sites being the terminal ileum and the ileocecal region (5, 6). On the basis of its morphological characteristics, it can be classified into cystic and tubular types and typically shows features such as the duplicated intestinal tube being adjacent to the normal intestinal tube on the mesenteric side; possessing a complete mucosal layer and smooth muscle structure,; and sharing the mucosal layer, mesentery, and blood supply system with the host intestinal tube (7). On the other hand, Meckel's diverticulum results from the abnormal regression of the yolk sac duct during development,which is typically located on the mesenteric opposite edge approximately 60–100 cm from the ileocecal region and is classified as a true diverticulum. It has full-thickness intestinal wall features (mucosal layer, muscular layer, and serosal layer)and is often accompanied by ectopic gastric mucosa or pancreatic tissue (8, 9).

In terms of imaging ultrasound and CT can clearly show the anatomical features of lesions, whereas 99mTc radionuclide imaging has high specificity for visualizing the ectopic gastric mucosa. Considering the specific circumstances of this case, what is unique about this case is as follows: (1) a young patient (requiring sedation for examination), (2) a prolonged course with deterioration of the overall condition, and (3) ultrasound suggesting intestinal blood supply impairment. Given the critical condition and signs of intestinal ischemia, we performed an emergency exploratory laparotomy even though we could not finish all the tests such as a CT scan. During the operation, considering the significant degree of abdominal distension and limited operative space, traditional open surgery was better for careful operations and checking the blood supply.

During the surgical process, to prevent missing any diagnoses, the surgeon needs to stay alert regarding the possibility of multiple lesions. The intestinal tube should be meticulously explored, especially in high-incidence areas such as the ileocecal region and terminal ileum, with a focus on examining both sides of the mesentery. Fortunately, in this clinical case, the Meckel's diverticulum was located only 30 cm proximal to the ileocecal valve, making the identification of the lesion relatively straightforward. However, surgeons must not discontinue their comprehensive exploration when a single pathological finding is discovered (10), with particular attention directed towards excluding the possibility of an inverted Meckel's diverticulum (11). Inversion of Meckel's diverticulum is a rare congenital intestinal malformation, with its pathological basis being the inversion of a diverticulum formed from incomplete regression of the yolk sac duct into the intestinal lumen. As intussusception caused by inverted Meckel's diverticulum is rare in children, its diagnosis is highly challenging: after intussusception is reduced due to local intestinal wall edema, the lesion often only presents as a depression in the intestinal wall, which can easily be misinterpreted as a simple postintussusception change. The characteristic manifestation of inverted Meckel's diverticulum is the presence of a micropore at the center of the depression, where a probe can be inserted into the apex of the inverted diverticulum, and even the diverticulum structure can be reduced, whereas the depression formed by intussusception compression is often hemispherical, with a smooth surface and no openings (12).

For symptomatic Meckel's diverticulum, current opinions are relatively clear. When patients exhibit mild inflammation or bleeding, conservative treatment can be initiated, including strict fasting (48–72 h), intravenous fluid therapy to maintain water and electrolyte balance, gastrointestinal decompression (nasogastric tube suction), and hemostatic agents (such as somatostatin analogues) combined with broad-spectrum antibiotics (covering G- bacteria and anaerobes) (13). However, if a patient develops mechanical intestinal obstruction, diverticulum perforation, or refractory bleeding, surgical treatment is needed. However, there are differing opinions on the management of incidentally discovered Meckel's diverticulum. A systematic review by Shermeen Rahmat et al. indicated that recent literature supports prophylactic resection in high-risk populations (aged <50 years, diverticulum length >2 cm, presence of ectopic tissue).They believe that the more criteria a patient meets, the greater the possibility that they will develop symptoms in the future (14). In our case, the child was young, with Meckel's diverticulum >2 cm, and postoperative pathology confirmed the observation of ectopic gastric epithelium at the top of the diverticulum. At our center, we advocate the following clinical protocol: After careful exclusion of contraindications (including but not limited to active peritonitis, malignancy, severe ascites, or immunocompromised status), diverticulectomy should be considered the preferred primary intervention. On the basis of our experience, when the nature of the lesions is clear, the patient's hemodynamics are stable, and the anastomosis technique meets standards. Simultaneous resection and anastomosis of multiple intestinal segments is also safe and feasible. Through meticulous surgical techniques and good perioperative management, the incidence of postoperative complications can be effectively reduced, thereby avoiding the possibility of reoperation.

There are several key takeaways from our experiences with cases of Intestinal Duplication-Induced Secondary Intussusception and Incidental Meckel's Diverticulum in an Infant treated at our center: (1) The diagnostic challenge of multiple lesions: Intestinal duplication malformations and Meckel's diverticulum often present as solitary cases in pediatric intestinal malformations, while multiple lesions are easily missed. The clinical manifestations of these two lesions are similar, and there is a lack of effective preoperative diagnostic methods to clarify the existence of multiple lesions. Therefore, meticulous exploration during surgery is key to diagnosing multiple lesions and reducing missed diagnoses. (2) The relationship between intussusception and intestinal malformations: Inversion of Meckel's diverticulum may serve as the starting point for secondary intussusception. After intussusception is reduced, careful differentiation is needed to determine whether there is a possibility of concurrent intestinal malformations. This step is crucial for a comprehensive assessment of the etiology and preventing the omission of potential lesions. (3) For incidentally identified Meckel's diverticulum, concurrent surgical intervention is the optimal choice in the absence of contraindications, and segmental resection of the affected bowel represents a safe and feasible therapeutic approach.During the surgical process, through meticulous operation and precise assessment, the incidence of postoperative complications can be effectively reduced, ensuring rapid recovery for patients. These insights remind us to maintain a high level of vigilance when handling such complex cases, to emphasize intraoperative exploration, and to flexibly apply surgical techniques to ensure the best treatment outcomes for patients.

## 4 Conclusions

In conclusion, while intestinal duplication and Meckel's diverticulum typically present as isolated pathological entities, their concurrent occurrence is exceedingly rare and prone to missed diagnosis. Notably, inverted Meckel's diverticulum—a potential lead point for secondary intussusception—underscores the critical role of meticulous intraoperative exploration as the gold standard for preventing diagnostic oversight. For incidentally identified Meckel's diverticulum, concurrent surgical intervention is the optimal strategy in the absence of contraindications, and segmental resection of the affected bowel represents a safe and feasible therapeutic approach to address coexisting pathologies.

### 4.1 Patient’s perspective

Patient's parents: Witnessing my child's full recovery after a single surgery that resolved both life-threatening intussusception and an unexpected Meckel's diverticulum fills me with immense gratitude. Although initial guilt and fears about undetected congenital risks lingered, the medical team's foresight to prevent future suffering transformed our crisis into hope. Today, my child's resilience and restored health remind me that compassionate, proactive care heals families as much as bodies do.

## Data Availability

The raw data supporting the conclusions of this article will be made available by the authors, without undue reservation.
